# Immunohistochemical testing of GISTs using CD117 markers: the UK NEQAS ICC & ISH external quality assessment data show significant differences in the performance of methods in regular use

**DOI:** 10.3389/bjbs.2026.16506

**Published:** 2026-07-17

**Authors:** Andrew Dodson, Suzanne Parry

**Affiliations:** UK National External Quality Assessment Scheme for Immunocytochemistry and In-Situ-Hybridisation (UK NEQAS ICC & ISH), London, United Kingdom

**Keywords:** CD117, EQA, GIST, immunohistochemistry, quality, external quality assessment

## Abstract

**Background:**

CD117 (c-KIT) is a tyrosine kinase receptor protein, mutations in which are important in the tumourigenesis of gastrointestinal stromal tumour (GIST). Immunohistochemical detection of CD117 is the primary identifying feature in its diagnosis.

**Introduction:**

UK NEQAS ICC & ISH conducts an EQA programme for CD117 expression in GIST. Between May-2020 and May-2025, 20 runs of this EQA were undertaken. The archived data was analysed to look for evidence regarding the quality of testing and associations between primary antibodies and stain quality.

**Discussion:**

Total submissions were 2,656 (mean per run = 132.8; range = 125–140). The number of submissions awarded a quality score indicating at least acceptable stain quality was 2,642 (99.5%). The total number of submissions that failed was 14 (mean proportion per run = 0.5%; range = 0.0%–2.4%); those obtaining a borderline score totalled 158 (mean proportion = 5.9%; range = 0.7%–10.9%); those obtaining an acceptable score totalled 394 (mean proportion = 14.8%; range = 4.3–24.4%); and those obtaining a good/excellent score totalled 2,090 (mean proportion = 78.7%; range = 57.7%–94.2%). There was a strong trend for the proportion of submissions obtaining a good/excellent quality score to increase over time. Four primary antibodies were used: Clone 9.7 (102 submissions, 3.9%), YR145 (263 submissions, 10.0%), EP10 supplied by Leica Biosystems (EP10(LB), 415 submissions, 15.8%), EP10 supplied by Roche Diagnostics (EP10(RD), 505 submissions, 19.2%), and a polyclonal antiserum supplied by Agilent Dako (1,263 submissions, 48.1%). A total of 76 submissions (2.9%) that used other primaries or suppliers were excluded from analysis.

**Conclusion:**

Demonstration of CD117 overexpression in GIST was undertaken to a very high quality standard in the majority of laboratories. For users of Dako Agilent supplied automation the highest quality staining was produced by using the polyclonal antiserum from the same supplier. Among users of Leica Biosystems and Roche Diagnostics automation the best staining was produced by using primary sourced from the same supplier that provided their automation. It is recommended that Clone 9.7 (Roche Diagnostics) should not be used as the results it produced were statistically significantly inferior to those of all other antibodies.

## Introduction

CD117 was first reported to be a sensitive marker of gastrointestinal stromal tumours (GISTs) by Miettinen *et al* at the end of the last century [1–3]. It continues to this day to be used as the primary marker in the diagnosis of this important mesenchymal tumour of the gastrointestinal tract [4].

GISTs arise predominantly from the interstitial cells of Cajal situated in the muscle layers of the bowel wall, with most presenting in the stomach or small intestine with either spindle cell or epithelioid morphology [5–9].

The *KIT* gene encodes CD117 (c-KIT) protein, which is a tyrosine kinase receptor. Mutations in the *KIT* gene are drivers of GISTs and also explain why they so commonly over-express the protein [10].

The introduction of treatments using tyrosine kinase inhibitors such as imatinib and sunitinib revolutionised the prognosis for patients with advanced unresectable or metastatic disease, it is therefore imperative that GISTs are correctly identified at primary diagnosis [11–13].

Despite its obvious importance, there are very few papers reporting on CD117 stain quality in the demonstration of GIST. One which is relevant was a report from 2006 [14]. In this study conducted by the College of American Pathologists they found that most laboratories that participated produced good quality staining. In their paper published in 2019 the NordiQC EQA organisation presented an analysis of the results from their CD117 quality assessments [15].

The UK National External Quality Assessment Scheme for Immunocytochemistry and In-Situ-Hybridisation (UK NEQAS ICC & ISH) [16] conducts quality assessment of the testing for GIST using CD117 immunohistochemistry in clinical laboratories in the UK and overseas. Its archives were surveyed, looking at the results from the last 5-years with the aim of producing evidence on the comparative performance of the immunohistochemical methods that are in clinical use.

## Materials and methods

The Scheme’s Alimentary Tract Pathology EQA Programme which looks at the immunohistochemical staining of markers for CD117 in GIST was examined. Assessment runs were conducted regularly between May 2020 and May 2025 (N = 20). Data on performance and immunohistochemical staining methodology were retrieved from the Scheme’s archives for each participant at each run.

### Participant characteristics

Participants in the EQA Programme were predominately healthcare-based cellular pathology departments undertaking immunohistochemical assessment of CD117 in the clinical setting. They were based in both the UK and overseas, with an almost equal split in frequency.

### EQA run design

Each participant was provided with a slide bearing unstained sections taken from formalin-fixed paraffin-embedded (FFPE) tissue samples. The samples comprised:

- Appendix, useful for assessing staining interstitial cells of Cajal and mast-cells both of which were expected to show specific staining, and for assessing non-specific staining elsewhere.
- A GIST, previously tested to ensure CD117 positivity.
- A relevant alternative tumour such as gastric adenocarcinoma, leiomyosarcoma or desmoid tumour, used to detected non-specific staining.

After undertaking immunohistochemical staining for CD117 using their in-house standard operating procedures, participants returned their stained slide to UK NEQAS ICC & ISH for collation and central visual assessment (see below for further details on the assessment process).

### Quality assessment metrics

The Scheme uses a scoring method at assessment that is based on four expert assessors evaluation. The four assessors score each participant’s submitted stained slide concurrently but independently using a multi-header microscope. Each assessor awards a score within a range of 1–5, with 1 being of no utility and 5 being excellent quality. These scores are summed for all four assessors to give a score in the range 4–20 (see Table 1 for further details).

**TABLE 1:** The Scheme’s qualitative scoring system.

Overall Quality Score	Individual Assessor Score	Interpretation
4–8	1	Unreadable. Staining has no diagnostic utility. Improvement essential
	2	Substantially sub-optimal preparation. Staining has no diagnostic utility. Improvement essential
12	3	Sub-optimal but readable preparation. The staining quality would allow the correct diagnosis to be achieved but requires substantial improvement
16–20	4	Good preparation. Staining appropriate for the target and of good quality. Only minor improvement(s) are possible
	5	Excellent preparation. Staining appropriate for the target and of excellent quality. No improvement required

Four assessors scored independently based on their visual assessment of the preparation’s stain quality. Assessor’s scores were only allowed to vary by one mark i.e., scores of 3 and 4 might be awarded by individual assessors scoring the same preparation, but not scores of 3 and 5; when scores between assessors did show such variations the quality of staining was discussed and a consensus was agreed. If an assessor wished to fail a participant’s submission i.e., award a score of 1 or 2, then all assessors must have agreed and awarded scores in the same range.

Samples were assessed against expected expression levels, which were established beforehand using preparations stained by the commercial suppliers of commonly used primary antibodies and in-house stained slides.

Sections, which had been cut at the same time as those distributed to participants were stained on the slide return deadline date to ensure the stability of expression.

To look for biological variation due to tumour heterogeneity sections taken at levels through the block were stained and assessed.

### Data analysis

Descriptive statistics were produced looking at trends in primary antibody usage over time.

The association of stain quality with key following elements of the immunohistochemical staining process were examined:

- Primary antibody (clone and supplier)
- Antigen retrieval (type, duration and supplier)
- Detection system (type and supplier)
- Automated platform (type and supplier)

The t-test (Mann-Whitney for non-parametric data) was used to assess level of significance (P < 0.05).

Data were collated and analysed using Excel (Office 365, Microsoft), which was also used for the preparation of charts and tables. Statistical analyses and box and whiskers chart production used the following software packages: SPSS (Version 29.0.2.0 (20), IBM) and Prism (Version 10.3.0, GraphPad).

## Results

In the period between May 2020 and May 2025 the Scheme conducted 20 assessment runs. The total number of submissions made was 2,656 (mean number of submissions per run = 132.8; range = 125–140). During the 5-year period of the study the number of participants rose from 125 to 140 (12.0% increase).

### Overall quality score results

The total number of submissions which were awarded a quality score indicative of at least acceptable stain quality (score ≥12/20, a “pass”) was 2,642 (99.5%).

The total number of submissions which failed assessment was 14 (score = 4–8; mean proportion per run = 0.5%; range = 0.0–2.4%), those obtaining a Borderline score totalled 158 (score = 12; mean proportion = 5.9%; range = 0.7–10.9%), for those obtaining an Acceptable the total was 394 (score = 13–15; mean proportion = 14.8%; range = 4.3–24.4%), and for those obtaining a Good or Excellent score the total was 2,090 (score = 16–20; mean proportion = 78.7%; range = 57.7–94.2%).

**FIGURE 1:**
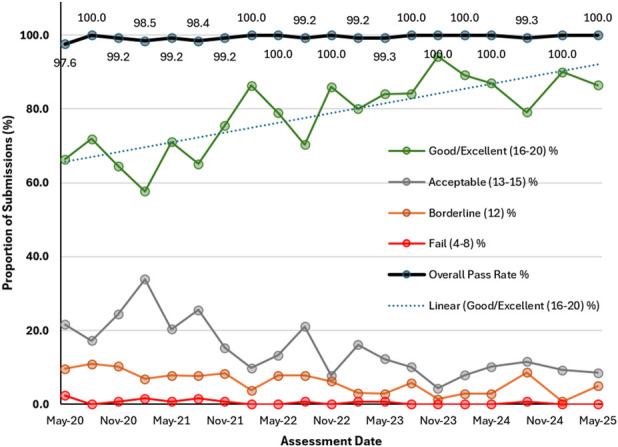
Descriptive statistics. The data shown in Table 2 are represented graphically here. For clarity data labels are presented for the overall proportions of submissions receiving a passing score only, for more details see Table 2. The blue-dotted line is the linear trendline for the proportion of submissions receiving a score indicative of a good or excellent stain quality (score 16–20).

There was a strong trend for the proportion of submissions which obtained a Good or Excellent quality score to increase over time. See Table 2; Figure 1 which show the data in detail.

**TABLE 2:** Descriptive statistics.

Run date	Fail (4–8)		Borderline (12)		Acceptable (13–15)		Good/Excellent (16–20)		Run total	
	N	%	N	%	N	%	N	%	N	% (*)
May-2020	3	2.4	12	9.6	27	21.6	83	66.4	125	4.7
Aug-2020	0	0.0	14	10.9	22	17.2	92	71.9	128	4.8
Nov-2020	1	0.8	13	10.2	31	24.4	82	64.6	127	4.8
Feb-2021	2	1.5	9	6.9	44	33.8	75	57.7	130	4.9
May-2021	1	0.8	10	7.8	26	20.3	91	71.1	128	4.8
Aug-2021	2	1.6	10	7.8	33	25.6	84	65.1	129	4.9
Nov-2021	1	0.8	11	8.4	20	15.3	99	75.6	131	4.9
Feb-2022	0	0.0	5	3.8	13	9.8	114	86.4	132	5.0
May-2022	0	0.0	10	7.8	17	13.3	101	78.9	128	4.8
Aug-2022	1	0.8	10	7.8	27	21.1	90	70.3	128	4.8
Nov-2022	0	0.0	8	6.3	10	7.8	110	85.9	128	4.8
Feb-2023	1	0.8	4	3.1	21	16.2	104	80.0	130	4.9
May-2023	1	0.7	4	2.9	17	12.3	116	84.1	138	5.2
Aug-2023	0	0.0	8	5.8	14	10.1	117	84.2	139	5.2
Nov-2023	0	0.0	2	1.4	6	4.3	131	94.2	139	5.2
Feb-2024	0	0.0	4	2.9	11	7.9	124	89.2	139	5.2
May-2024	0	0.0	4	2.9	14	10.1	120	87.0	138	5.2
Sep-2024	1	0.7	12	8.6	16	11.5	110	79.1	139	5.2
Jan-2025	0	0.0	1	0.7	13	9.3	126	90.0	140	5.3
May-2025	0	0.0	7	5.0	12	8.6	121	86.4	140	5.3
Total	14	​	158	​	394	​	2090	​	2656	​
Mean	​	0.5	​	5.9	​	14.8	​	78.7	​	100.0

Showing for each Run the number (N) and proportion (%) of submissions assessed to be in each quality score category, together with the total number of submissions at that Run. The figure shown in the Run Totals column indicated by the ‘*’ symbol is the proportion of the total submissions. See also Figure 1.

### Primary antibodies

Details on the primary antibodies used were returned for 2,624 submissions (98.8%).

Four rabbit monoclonal and two rabbit polyclonal reagents were used to detect CD117. Three of the four monoclonals (Clone 9.7, DBRRM1.17 and YR145) were each obtained from a single different commercial supplier respectively, while EP10 was sourced from five different suppliers. The two polyclonal antisera were each supplied by two different suppliers. See Table 3 for details and references.

**TABLE 3:** Primary antibodies: descriptive statistics.

Primary Antibody and Supplier	Product Code	Type	Format	Count (N)	Proportion (%)
Clone 9.7 [17]	​	​	​	102	3.9
Roche diagnostics	790–2951	Rb Mab	RTU	102	**3.9**
DBRRM1.17 [18]	​	​	​	**5**	**0.2**
Diagnostic biosystems	RMAB119	Rb Mab	RTU	5	**0.2**
EP10 [19–21]	​	​	​	**982**	**37.4**
Biocare Medical	PME-296AA	Rb Mab	Concentrate	18	**0.7**
CliniSciences	MAD-000644QD	Rb Mab	RTU	1	**0.0**
Epitomics	AC-0029	Rb Mab	Concentrate	7	**0.3**
Roche diagnostics	790–7061	Rb Mab	RTU	505	**19.2**
Leica biosystems	CD117-032-L-CE	Rb Mab	Concentrate	36	**1.4**
Leica biosystems	PA0007	Rb Mab	RTU	415	**15.8**
Polyclonal [22, 23]	​	​	​	**1,272**	**48.5**
Agilent dako	A4502	Rb pab	Concentrate	1,263	**48.1**
Generon	PDR045	Rb pab	Concentrate	9	**0.3**
YR145 [24, 25]	​	​	​	**263**	**10.0**
Cell Marque	117R-18	Rb Mab	Concentrate	263	**10.0**
Total	​	​	​	**2624**	**100.0**

Details of the primary antibody reagents used. Showing both the aggregated data for each primary antibody reagent and the breakdown by supplier and format. For 32 submissions the participant did not supply methodological data. Rb Mab = rabbit monoclonal; Rb Pab = rabbit polyclonal; RTU = ready-to-use. Bold values indicate summations for that category.

The primary antibody reagents were supplied as either prediluted ‘ready-to-use’ (RTU) or concentrated formulations. Overall the total number of submissions that used RTU reagents was 1,028 (39.2%), while those using a diluted solution taken from a concentrate totalled 1,596 (60.8%). Table 3 shows this and additional descriptive data for the primary antibodies.

In order to present the significant results clearly, individual antibody reagent/supplier combinations represented at a frequency of <100 submissions (<3.9%) were excluded from further discussion. The number of submissions for the six reagent/supplier combinations that were excluded had a range of 1–36 (mean: 12.6), thus even the most prevalent of them was used by less than two participants at any given run. Any conclusions derived from analysis of their data would have been entirely anecdotal.

Five primary antibody reagents will be discussed: Clone 9.7 (102 submissions, 3.9%), YR145 (263 submissions, 10.0%), EP10 supplied by Leica Biosystems (EP10(LB), 415 submissions, 15.8%), EP10 supplied by Roche Diagnostics (EP10(RD), 505 submissions, 19.2%), and a polyclonal antiserum supplied by Agilent Dako (1,263 submissions, 48.1%). In total 76 submissions (2.9%) were excluded from the subsequent analyses.

### Primary antibody performance

The analysis of quality score data indicated that the demonstration of CD117 in the UK NEQAS provided materials was good. For the whole population the mean quality score was 16.3 (95% CIs: 16.1–16.4).

### Primary antibody usage

**FIGURE 2:**
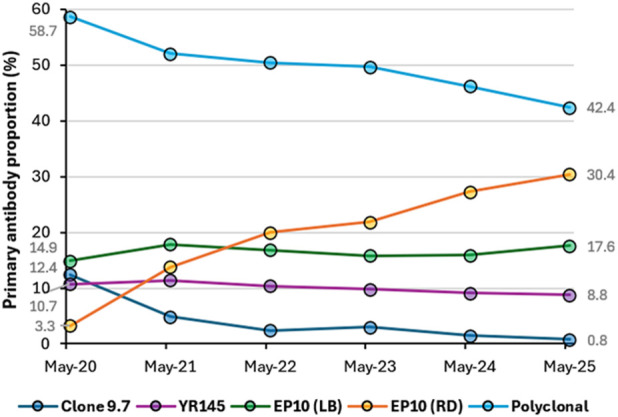
Trends in proportional use of primary antibodies over time. The change proportion of submissions using each of the primary antibody reagents is shown per year.

the use of the primary antibody reagents over the 5-year period of the study was examined, with consistent trends been seen. The proportional use of the polyclonal antiserum declined from 58.7% in 2020 to 42.4% in 2025. There was also a decline in the use of the 9.7 clone from 12.4% to 0.8%, and the YR145 clone from 10.7% to 8.8%. In contrast the EP10 clone showed an increase in the proportion of users from 18.2% (3.3% for the Roche Diagnostics supplied reagent and 14.9% for the Leica Biosystems supplied reagent) to 48.0% (30.4% for EP(RD) and 17.6% for EP(LB)), such that the EP10 clone became the most frequently used primary reagent at any given assessment during the course of 2025, overtaking the polyclonal antiserum which had consistently held that position previously. See Figure 2, which illustrates these data.

### Factors associated with fails at assessment

Overall, 12 submissions (12/2548, 0.5%) were assessed as fails (quality score = 8/20). When dealing with such a small dataset it is not possible to drawn any firm conclusions about factors associated with assay failure. However, it is worthwhile to note that the assessors assigned only two indications to failed submissions, the first being very weak staining of the GIST sample and normal elements expected to stain i.e., interstitial cells of Cajal and mast cells which was noted in seven submissions (7/12, 58.3%), and the second was excessive non-specific and/or inappropriate staining particularly in the non-GIST tumour and the appendix samples, which was noted for five submissions (5/12, 51.7%).

The polyclonal reagent was used in eight of the failed submissions (8/12, 75.0%), but the preponderance is not surprising given that the polyclonal antiserum was used in just under half of all submissions made (1,263/2548, 49.6%) and was present in the data set more than twice as frequently as the next most “popular” antibody (EP10(RD), 505/2548, 19.8%). See also Table 4.

**TABLE 4:** Factors present in submissions that failed at assessment.

Run	Assessors’ Comments	Antibody (dilution)	Antigen Retrieval	Detection System	Automation
133	False negative	Clone 9.7 (RTU)	NS	NS	NS
130	False negative	Polyclonal (1:500)	High pH TRS	FLEX+	BenchMark ULTRA
141	False negative	Polyclonal (NS)	CC1	Optiview	Ventana
130	False negative	YR145 (1:50)	NS	NS	NS
136	Non-specific/Background	EP10(RD) (RTU)	NS	NS	NS
135	Non-specific/Background	Polyclonal (1:100)	High pH TRS	FLEX+	Omnis
139	Non-specific/Background	Polyclonal (1:200)	ER2	BOND refine	BOND-III
134	Non-specific/Background	Polyclonal (1:300)	ER2	BOND refine	BOND-III
147	Non-specific/Background	YR145 (NS)	CC1	UltraView	BenchMark ULTRA
133	Very weak	Polyclonal (NS)	CC1	Optiview	BenchMark ULTRA
130	Weak	Polyclonal (1:1,000)	CC1	UltraView	BenchMark ULTRA
132	Weak	Polyclonal (1:200)	High pH TRS	FLEX+	Omnis

RTU = ready to use, NS = not stated, CC1 = cell conditioning reagent 1 (high pH), TRS = target retrieval solution, ER2 = epitope retrieval buffer (high pH).

### Association of primary antibody and automation

There were clear difference in the degree to which primary antibody and automation supplier were associated. For Roche Diagnostics there was an absolute association for both Clone 9.7 and EP10(RD), and for Leica Biosystems it was extremely high (94.9%) for the EP10(LB) reagent. In contrast the polyclonal antiserum supplied by Agilent Dako was used on machines manufactured by the same supplier with a frequency of only 26.7%. Indeed it was used more frequently on both Leica Biosystems (27.6%) and Roche Diagnostics supplied automation (45.8%).

The YR145 clone (supplied by Cell Marque) was used primarily in conjunction with Roche Diagnostics supplied automation (82.5%). See also Table 5.

**TABLE 5:** Association of Primary antibody supplier and Automation supplier.

Primary antibody clone	Primary antibody supplier	Agilent Dako		Leica biosystems		Roche diagnostics		Totals
		N	%	N	%	N	%	N
Clone 9.7	Roche diagnostics	0	0.0	0	0.0	102	100.0	102
EP10(RD)	Roche diagnostics	0	0.0	0	0.0	505	100.0	505
EP10(LB)	Leica biosystems	20	4.8	393	94.9	1	0.2	414
Polyclonal	Agilent dako	337	26.7	348	27.6	578	45.8	1,263
YR145	Cell Marque	27	10.3	19	7.2	217	82.5	263
Totals	​	384	15.1	760	29.8	1,403	55.1	2547

Correlating the supplier of the primary antibody with the supplier of the automation it was used on. The grey-shaded boxes highlight the matched combinations. N = count of submissions, % = proportion of submission. Data for one submission has been omitted as the automation type was not submitted.

**FIGURE 3:**
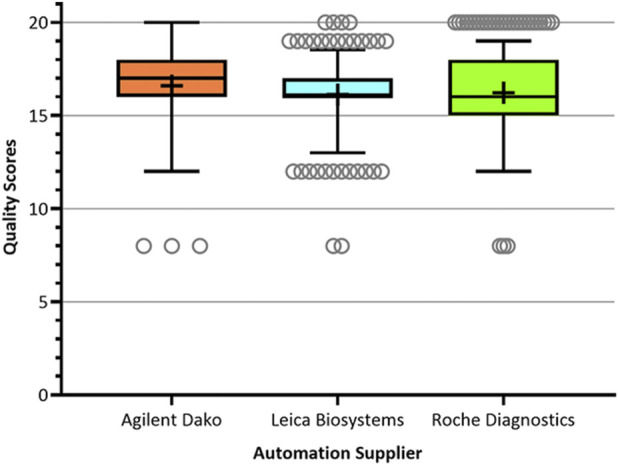
Box and whiskers plot illustrating quality score distributions for polyclonal primary antibody used on different automated platforms. Box heights indicate the 25% and 75% interquartile range, the whiskers show the 5% and 95% percentiles. The lines within boxes show the median and the crosses the mean quality scores.

In light of the use of the polyclonal antiserum across all major suppliers’ platforms, a situation which differed markedly from that seen in the use of both EP10(LB) and EP10(RD), an analysis was undertaken to look for an association of performance with individual platform supplier. The mean quality score was highest when the polyclonal primary was used on Agilent Dako machines (16.6, 95% CIs: 16.4–16.8), when used on the Leica Biosystem machines it was 16.1 (95% CIs: 16.0–16.3), and on Roche Diagnostic supplied machines it was 16.2 (95% CIs: 16.1–16.4). See Figure 3.

The difference in quality score distributions was statistically significant when comparing Agilent Dako and Leica Biosystems (P < 0.0001) and Agilent Dako and Roche Diagnostics (P = 0.0013). It was not different when Leica Biosystems and Roche Diagnostics machines were compared (P = 0.216).

**FIGURE 4:**
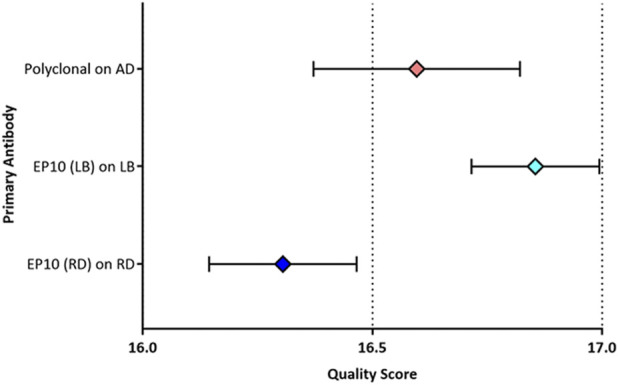
Forest plot illustrating quality score distributions for primary antibodies and matched automated platforms. The diamond shaped boxes indicate the mean quality scores; the whiskers show the 95% confidence intervals. AD = Agilent Dako, LB = Leica Biosystems, RD = Roche Diagnostics.

A follow-on analysis looked at the results for submissions which used primary antibody reagent and the automation matched for supplier. It found that EP10(LB) on Leica Biosystems machines and the polyclonal antiserum on Agilent Dako machines were both associated with superior quality staining results compared to those produced by EP10(RD) on Roche Diagnostics machines. But they were not statistically different from each other. See Figure 4; Table 8.

## Discussion

The recent data produced by UK NEQAS ICC & ISH in its CD117 in GIST EQA Programme was analysed to look for evidence on the performance of the primary antibody reagents that are used and their effect on the quality of staining produced. The main aim was to produce robust evidence-based information that could be used by those undertaking clinical testing to ensure they are achieving the highest quality results possible, and in so doing assure diagnostic outcomes.

The data available to us was drawn from immunohistochemistry methodology records that accompanied the stained slides submitted over the course of twenty GIST EQA assessment runs that were conducted over a 5-year period between May 2020 to May 2025. During that time the Scheme assessed the quality of 2,656 submissions and received data on the methodology used for 2,624 of them. Having such large datasets made it possible to find associations between the quality and the method that were highly statistically significant.

The overall proportion of submission receiving at least a borderline acceptable quality score was 99.5% with the lowest at any run being 97.6%, indicating that this marker was demonstrated to an adequate standard in the vast majority of laboratories. However this does not mean there was no room for improvement, as is indeed reflected in the proportion of submissions receiving a score indicating good or excellent staining, which is the standard all laboratories should be aiming to achieve. This was 78.4% on average (range 57.7%–94.2%) i.e., at one point only just over half of submissions demonstrated good or excellent staining.

Our survey has captured a period over which a dynamic change in the quality of staining occurred, as can be clearly appreciated when the data presented in Figure 1 are reviewed. There was a noticeable sustained increase in the proportion of submissions showing good or excellent staining from 65% at the first run in 2020 to 90% in the last in 2025. It is probable that a major proportion of the improvement can be attributed to the concomitant change in the landscape of primary antibody usage that occurred, this will be discussed in more detail below.

Four primary antibody clones and one polyclonal antiserum were reported as being used, and as is usually seen these were supplied by one or other of the three main commercial suppliers in the field, namely Agilent Dako, Leica Biosystems and Roche Diagnostics. There was one notable exception in the YR145 clone that was supplied by Cell Marque. By not including them in the analyses no disrespect is intended to companies which supplied antibodies that were only used on a small number of occasions, the necessity to make evidence-based associations precluded them.

Historically, the polyclonal antiserum (Agilent Dako, A4502) dominated the field, being the first commercially available primary to be widely reported as being effective in the demonstration of CD117 in formalin-fixed paraffin embedded (FFPE) samples. In this study submissions using it represented just less than half of the total. However, it is notable that its proportional use showed a steady decline over the course of the study (from 58.7% to 42.4%), such that the monoclonal primaries when taken in aggregate now hold the larger share of the market.

The rabbit monoclonal antibody EP10, available from a variety of suppliers but notably in our data from Leica Biosystems as an RTU (PA0007) and Roche Diagnostics also as an RTU (790–2951) saw an increase in their use. The Roche Diagnostics supplied reagent hereafter referred to as EP10(RD) saw a ten-fold increase proportional use from 3.3% to 30.4%, such that it became the second-most commonly used primary antibody. For the most-part the increase in use was due to laboratories that had previously used the rabbit polyclonal antiserum changed to using EP10(RD), and this was particularly prevalent amongst laboratories that used Roche Diagnostic automation. Taking EP10(RD) and the Leica Biosystems supplied reagent (EP10(LB)) together, the proportion of EP10 users was larger than that of the polyclonal antiserum at the time the survey period ended (48.% compared to 42.4% respectively).

A third notable change in primary antibody use was the decline in use of the rabbit monoclonal antibody 9.7 (Roche Diagnostics, RTU, 790–2951), which had a 12.4% share of users among participants in 2020 that fell to just 0.8% in 2025. Again this was due principally to participants changing to EP10(RD).

When the performance of the primaries was examined using higher quality score as the indicator of superior performance the performance of the EP10(LB) reagent was 16.9 (95% CIs: 16.7–17.0). No submission that used this primary failed at assessment, which was in contrast to all other primary reagents where there was at least one fail. Its performance was superior to that of all other primary reagents, with the difference being significant at P < 0.05 when compared to Y145 and highly significant (P < 0.0001) when compared to both EP (RD) and the polyclonal antiserum.

Submissions that used Clone 9.7 had the worst performance with a mean quality score of 14.6 (95% CIs: 14.2–15.0). There was a highly significant association between the use of this antibody clone and lower quality scores (P < 0.0001), when its performance was compared to all other reagents.

**FIGURE 5:**
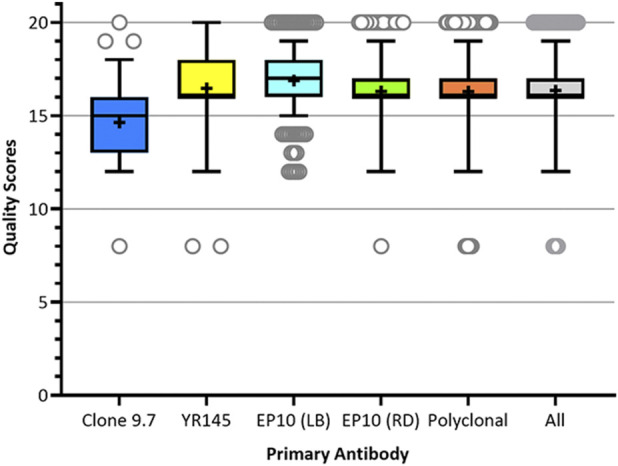
Box and whiskers plot illustrating quality score distributions for primary antibodies. Box heights indicate the 25% and 75% interquartile range, the whiskers show the 5% and 95% percentiles. The lines within boxes show the median and the crosses the mean quality scores.

The Y145, the EP (RD) and the polyclonal antiserum produced results that were not significantly different from one another. See Table 6; Figure 5, which show antibody performance data in detail and Table 7 which shows the statistical significance analysis results.

**TABLE 6:** Primary antibodies: quality score analysis.

Descriptive statistic	Clone 9.7	YR145	EP10 (LB)	EP10 (RD)	Polyclonal	All
Count (N)	102	263	415	505	1,263	2548
25% percentile	13.0	16.0	16.0	16.0	16.0	16.0
Median	15.0	16.0	17.0	16.0	16.0	16.0
75% percentile	16.0	18.0	18.0	17.0	17.0	17.0
Mean	14.6	16.5	16.9	16.3	16.3	16.3
Std. Deviation	2.1	2.1	1.4	1.8	1.9	1.9
Lower 95% CI	14.2	16.2	16.7	16.1	16.2	16.2
Upper 95% CI	15.0	16.7	17.0	16.5	16.4	16.4

Details of the primary antibody performance. Std. = Standard; CI = confidence interval of the mean.

**TABLE 7:** Results of test to compare quality scores.

Primary antibody	Clone 9.7	Y145	EP10 (LB)	EP10 (RD)	Polyclonal
Clone 9.7	​	<0.0001	<0.0001	<0.0001	<0.0001
Y145	****	​	0.0250	0.1925	0.0902
EP10 (LB)	****	*	​	<0.0001	<0.0001
EP10 (RD)	****	NS	****	​	0.8094
Polyclonal	****	NS	****	NS	​

The Mann-Whitney test compares the distributions of two unmatched groups at P < 0.05. * = different at P < 0.05; **** = different at P < 0.0001; NS = not significantly different.

As was outlined in the Results section a markedly different pattern was found when the data for primary antibody usage across the machines of the three major automated platform suppliers was examined. It was clear that antibody and platform was very closely coupled for clone 9.7, EP10(LB) and EP10(RD). But this was not the case for the polyclonal reagent from Agilent Dako; the proportion of its users that used it on Agilent Dako sourced automation was actually smaller than the proportions which used it on Leica Biosystems or Roche Diagnostics machines. The data was further examined to see if this affected performance results for the polyclonal antiserum and found that it did. The size of the effect was such that the performance of the polyclonal antiserum when it was used on Agilent Dako automated platforms became equivalent to the best performing antibody, EP10(LB) (no significant difference at P < 0.05) (See Table 8; Figure 3).

**TABLE 8:** Results of test to compare quality scores.

Primary antibody (Supplier)	EP10 (LB) on LB	EP10 (RD) on RD	Polyclonal on AD
EP10 (LB)	​	<0.0001	0.3425
EP10 (RD)	****	​	0.0084
Polyclonal	NS	**	​

Mann-Whitney test to compares the distributions of two unmatched groups at P < 0.05. ** = different at P < 0.005; **** = different at P < 0.0001; NS, not significantly different.

This indicates the importance of analysing the data in a context sensitive way and one that reflects real-world usage.

This work represents an advance in biomedical science because it uses a set of real-world data to produce evidence that can be used to assure laboratory testing quality and improve patient outcomes.

### Conclusions

The demonstration of CD117 over-expression in GIST is undertaken to a very high standard of quality in the majority of laboratories that participate in regular scheduled EQA for this important predictive biomarker.

Statistically tested and proven results indicate that for users of Dako Agilent supplied automation the highest quality staining is produced by using the polyclonal antiserum from the same supplier.

Users of Leica Biosystems and Roche Diagnostics automation should use the EP10 rabbit monoclonal primary, and should source that reagent from the same supplier that provides their automation.

It is recommended that Clone 9.7 as supplied by Roche Diagnostics should not be used as the results it produces are statistically significantly inferior to those obtained using all other surveyed primary antibodies.

## Summary table

### What is known about this subject:

- CD117 (c-KIT) is an important marker that is used in the diagnosis of gastrointestinal stromal tumours (GISTs).
- Mutational analysis of the *KIT* gene is used to guide therapy choice following a confirmed diagnosis of GIST.
- Targeted treatment using imatinib, which is a tyrosine kinase inhibitor, is highly effective in approximately 85% of GISTs.

### What this paper adds:

- This work presents information to aid in the choice of immunohistochemical methods that optimise testing quality.
- The results confirm that the majority of laboratories perform testing for CD117 to a high standard.
- The choice of primary antibody should be guided by the type of automation available to the laboratory.

## Data Availability

The data analyzed in this study is subject to the following licenses/restrictions: confidential quality assessment data. Requests to access these datasets should be directed to adodson@ukneqasiccish.org.
